# APE1 promotes non-homologous end joining by initiating DNA double-strand break formation and decreasing ubiquitination of artemis following oxidative genotoxic stress

**DOI:** 10.1186/s12967-023-04022-9

**Published:** 2023-03-09

**Authors:** Qin Zhang, Lujie Yang, Han Gao, Xunjie Kuang, He Xiao, Chen Yang, Yi Cheng, Lei Zhang, Xin Guo, Yong Zhong, Mengxia Li

**Affiliations:** Cancer Center of Daping Hospital, Army Medical University, Chongqing, 400000 China

**Keywords:** DNA-PK_cs_, DNA damage response, Radio-resistance, ATM

## Abstract

**Background:**

Apurinic/apyrimidinic endonuclease 1 (APE1) imparts radio-resistance by repairing isolated lesions via the base excision repair (BER) pathway, but whether and how it is involved in the formation and/or repair of DSBs remains mostly unknown.

**Methods:**

Immunoblotting, fluorescent immunostaining, and the Comet assay were used to investigate the effect of APE1 on temporal DSB formation. Chromatin extraction, 53BP1 foci and co-immunoprecipitation, and rescue assays were used to evaluate non-homologous end joining (NHEJ) repair and APE1 effects. Colony formation, micronuclei measurements, flow cytometry, and xenograft models were used to examine the effect of APE1 expression on survival and synergistic lethality. Immunohistochemistry was used to detect APE1 and Artemis expression in cervical tumor tissues.

**Results:**

APE1 is upregulated in cervical tumor tissue compared to paired peri-tumor, and elevated APE1 expression is associated with radio-resistance. APE1 mediates resistance to oxidative genotoxic stress by activating NHEJ repair. APE1, via its endonuclease activity, initiates clustered lesion conversion to DSBs (within 1 h), promoting the activation of the DNA-dependent protein kinase catalytic subunit (DNA-PK_cs_), a key kinase in the DNA damage response (DDR) and NHEJ pathway. APE1 then participates in NHEJ repair directly by interacting with DNA- PK_cs_. Additionally, APE1 promotes NHEJ activity by decreasing the ubiquitination and degradation of Artemis, a nuclease with a critical role in the NHEJ pathway. Overall, APE1 deficiency leads to DSB accumulation at a late phase following oxidative stress (after 24 h), which also triggers activation of Ataxia-telangiectasia mutated (ATM), another key kinase of the DDR. Inhibition of ATM activity significantly promotes synergistic lethality with oxidative stress in APE1-deficient cells and tumors.

**Conclusion:**

APE1 promotes NHEJ repair by temporally regulating DBS formation and repair following oxidative stress. This knowledge provides new insights into the design of combinatorial therapies and indicates the timing of administration and maintenance of DDR inhibitors for overcoming radio-resistance.

**Supplementary Information:**

The online version contains supplementary material available at 10.1186/s12967-023-04022-9.

## Background

Radiotherapy is one of the cornerstones of cancer treatment [1]. Ionizing radiation (IR) induces tumor cell death by generating a range of DNA damage forms, which are broadly categorized as isolated or clustered DNA lesions based on distance between the modifications [2, 3]. Isolated DNA damage, including single strand breaks (SSBs), oxidized bases and AP sites, which are located typically more than a helical turn from one another, are mostly repaired efficiently. Conversely, clustered lesions, consisting of closely spaced DNA modifications (generally defined as within 10 bp), have long been postulated to be the lethal damage induced by IR. At present, radio-resistance is still a major limitation for radiotherapy applications, and a more comprehensive understanding of the molecular processes related to IR-induced DNA damage in tumor cells is needed to eventually overcome tumor radio-resistance.

It is well established that approximately 70% of IR-induced DNA damage is formed by reactive-free radicals (ROS) produced by the radiolysis of water in the vicinity of DNA [4, 5]. The base-excision repair (BER) pathway, an evolutionarily conserved process, is essential for the repair of isolated DNA lesions induced by various damaging agents, particularly oxidizing agents [6, 7]. Apurinic/apyrimidinic endonuclease 1 (APE1) is a key enzyme of the BER pathway, and the lack of an effective back-up endonuclease or repair mechanism results in early embryonic lethality in mice that lack the APE1 gene [8]. In BER, APE1 primarily functions to hydrolyse the phosphodiester backbone immediately 5 ´ to an AP site following removal of a damaged base by a DNA glycosylase, thereby facilitating the full response of the other BER factors and promoting repair [9]. Meanwhile, APE1 also functions as a transcription factor regulator during conditions of oxidative stress via its so-called redox effector factor (REF-1) activity [10]. Multiple pre-clinical studies have shown that elevated APE1 expression or activity is associated with resistance to chemo- or radiotherapy, and some clinical studies also report that APE1 is upregulated in numerous cancers. Collectively, it has been postulated that APE1 represents a promising therapeutic and prognostic molecular target [11, 12].

In the primary work carried out to date, the mechanism by which APE1 mediates therapeutic agent resistance is thought to involve resolution of isolated toxic DNA damage. The main emphasis has therefore been to inactivate APE1 activity in combination with a relevant genotoxin, such as an alkylating agent that creates BER substrates, to enhance therapeutic efficacy [13]. However, a randomized, double-blind, placebo-controlled study led by our group showed that an APE1 endonuclease inhibitor combined with docetaxel and cisplatin failed to improve the overall survival (OS) of advanced non-small cell lung cancer patients exhibiting high expression of APE1 compared to chemotherapy alone, indicating there are still unknown resistant mechanisms [14].

DNA double-strand breaks (DSBs) are thought to be the most-deleterious IR-induced clustered DNA damage [15]. In vitro studies using purified enzymes demonstrated that if two lesions are located at least three nucleotides apart on opposed strands, APE1 is capable of recognizing and cleaving both lesions giving rise to a DSB [16]. Thus, it seems likely that APE1 is not only responsible for repairing isolated DNA lesions via the BER pathway but may also affect DSB levels by processing clustered DNA substrates. Whether and how APE1 is involved in the formation and/or repair of DSBs under the stress of IR and other oxidative damage in vivo remains mostly unknown.

In the studies here, we aimed to determine the role of APE1 in DSB formation and/or repair in response to oxidative genotoxic stress in human tumor cells. We report that APE1 regulates therapeutic resistance under oxidative stress by activating and participating in non-homologous end joining (NHEJ) repair, both indirectly and directly. Specifically, APE1 via its endonuclease activity converts clustered lesions into DSBs (within 1 h) following oxidative stress, which is a prerequisite for the subsequent NHEJ repair process. In addition, APE1 decreases the ubiquitination and degradation of Artemis, a nuclease with a critical role in the NHEJ pathway. Combined, the data underscore the importance of APE1 levels in dictating therapeutic agent resistance, both in terms of genotoxic damage formation and DNA repair, insights that have relevance to the timing of administration and maintenance of DNA repair inhibitors during radiotherapy.

## Methods

### Cell culture and chemical reagents

HeLa, SiHa WT cell lines were purchased form National Collection of Authentical Cell Cultures of China. HeLa C65S-APE1 and HeLa WT-APE1 (provided by Gianluca Tell Lab, Department of Medical and Biological Sciences, University of Udine, Udine, Italy). Cells were cultured in DMEM medium (Gibco, China), supplemented with 1% Penicillin/Streptomycin (Hyclone, US) and 10% fetal bovine serum (Gibco, US) and were monitored for mycoplasma contamination using the Mycoplasma PCR Detection Kit (Beyotime, catalog No.C0301S, Shanghai, China). All chemical reagents were supplied by Selleck (Shanghai, China) unless otherwise specified mentioned. E3330 (catalog No. S7445), Ku55933 (catalog No. S1092), MMS (catalog No. E0609), MG132 (catalog No. S2619), Doxycycline (catalog No. S5159). Cycloheximide (catalog No.C4859, Sigma, USA), Inhibitor III (Sigma, catalog No.262017, USA), TBHP (Sigma, catalog No.416665, USA). The 8MV X-rays at indicated doses were generated by (Elekta, synergy 2178).

### Biological resources

The Artemis siRNA (5′-GCAUUAAGCCAUCCACCAUTT-3′), the vector of flag-tagged Artemis and flag-tagged negative control (NC) come from Hanbio Tech Co. Ltd. (Shanghai, China). The flag-tagged Artemis and NC cDNA were cloned into the empty vector pcDNA3.1-3xflag (Hanbio, Shanghai, China). For APE1 knockdown assays, two different shRNAs were utilized to generate stable APE1 knockdown cell lines. The knockdown hairpin sequences of APE1 are 5′- GATCCCCCCTGCCACA-CTCAAGATCTGCTTCAAGAGAGCAGATCTTGAGTGTGGCAGGTTTTTGGAAA-3′ (Jikai, Shan-ghai, China) and 5′-CCGGCAGAGAAATCTGCATTCTATTCTCGAGAATAGAATGCAGATTTCTC-TGTTTTT-3′ (clone numbers: TRCN0000007958, Sigma, St. Louis, MO, USA). Then lentiviral were transduced into the HeLa and SiHa cell lines and selected with 4 μg/ml puromycin. For inducible silencing of endogenous APE1 and reconstitution with WT- APE1 and C65S-APE1 proteins, doxycycline was added to the cell culture medium at the final concentration of 1 μg/ml, and cells were grown for 14 days [17]. Primers of Artemis used for RT-PCR are listed as followed. Forward: 5′AGTACGGAGCCAAAGTATAAACCACT3′, Reverse: 5′TCCGGGTATGGAACTTTGT-GC3′.

### Immunoblotting, fluorescent immunostaining and co-immunoprecipitation

The following commercial antibodies were used to immunoblotting: anti-phospho-H2AX (S139) (EMD Millipore, 05–636), anti-H2AX (Cell Signaling Technology, 7631), anti-DNA-PK_cs_ phospho-S2056 (Abcam, ab124918), anti-DNA-PK_cs_ (Abcam, ab70250), Ku80 (Santa Cruz, sc5280), Ku70 (Santa Cruz, sc17789), anti-APE1(Abcam, 189474), anti-APE1(Abcam, ab192), anti-ATM phospho-S1981 (Abcam, ab81292), anti-KAP1 phospho-S824 (Abcam, ab70369), anti-LIG4 antibody (Cell Signaling Technology, 14649), cleaved-PARP(ab32064), cleaved-Caspase3(ab32042), Artemis (Cell Signaling Technology,13381), Artemis(Invitrogen, PA5-102814), Ubb (Cell Signaling Technology, 3936), Anti-DDDDK tag (Abcam, ab1162). Secondary antibodies included anti-mouse IgG (HRP-linked) and anti-rabbit IgG (HRP-linked), which were purchased from Bio-Rad. anti-tubulin (Cell Signaling Technology, 2144), anti-actin (Abcam, ab8227), anti-Histone H3 antibody (Cell Signaling Technology, 9715). The following commercial antibodies were used to fluorescent immunostaining: γ-H2AX (EMD Millipore, 05–636) or 53BP1 (Santa Cruz, sc517218) or Cyclin A2 (Abcam, ab181591). Secondary antibodies included Fluor 488 (Cell Signaling Technology, 4412) and Alexa Fluor 562 (Cell Signaling Technology, 8889S). The following commercial antibodies were used to co-immunoprecipitation: anti-APE1 (Abcam, ab192). Immunoblotting [18], fluorescent immunostaining [19] analysis and co-immunoprecipitation [20] was performed following procedures described previously. All quantified data are derived from at least three independent experiments.

### Colony formation assay

Harvest exponentially growing cells and re-plate on 60 mm dishes. Then cells were mock treated or treated as indicated in figures. Leave the dishes in the incubator for 14 days. Cells were fixed and stained with 100% methanol solution containing 0.1% crystal violet. Colonies were scored and the mean value for triplicate culture dishes was determined. Cell survival was normalized to plating efficiency of untreated controls for each cell type [21].

### Alkaline/neutral comet assay

For the alkaline/neutral comet assay, HeLa NC and shAPE1 cells were mock treated or treated as indicated in figures, alkaline/neutral Comet assay was performed according to the manufacturer’s protocol (Trevigen, Catalog 4250-050-K). CaspLab was used for automated analysis of the comets. At least 100 comets for each condition were analyzed for plotting the comet tail moment data and statistical analyses.

### Subcellular fractionation

The recruitment of NHEJ proteins to chromatin following TBHP-induced was examined using the Subcellular Protein Fractionation Kit (Thermo Fisher, 78840). Briefly, the HeLa NC and shAPE1 cells were mock treated or 100 μM TBHP-treated 1 h and allowed to recover for 1 h and 4 h. Next, the cells were harvested after trypsinization and processed with the Thermo Fisher Subcellular Protein Fractionation Kit according to the manufacturer’s instructions.

### Immunohistochemistry (IHC) staining

All assay about human samples were performed with the approval of the Ethics Committee of Army Medical Center of People’s Liberation Army. 46 cervical cancer patient samples with FIGO stage IIB-III and received radical chemo-radiotherapy and 16 with FIGO stage I-IIA stage and received radical surgery in Daping Hospital of 2017–2018 were included in this study for evaluated the expression level of APE1 and Artemis. The following commercial antibodies were used to IHC: anti-APE1 (Abcam, ab189474) or anti-Artemis (Cell Signaling Technology,13381). The IHC analysis was performed following procedures described previously [22].

### Micronuclei detection

Seed cells (2 × 10^5^cells) in coverslips 1 day before the experiment. The cells were mock- or treated as indicated in figures and then changed with fresh medium to recover for 4 days. Briefly, after fixing, the cover slip was mounted in VectaShield Antifade Mounting Medium Containing 4′,6-diamidino-2-phenylindole (LsBio, J1033). The images were acquired using the Imager fluorescence microscope (Nikon, DS-Qi2) utilizing a 40 × objective lens. At least 100 cells for each condition were analyzed and counted for plotting and statistical analyses.

### Apoptosis and cell cycle analysis

Cells were seed (2 × 10^5^cells) in a 6 well plate 1 day before the experiment. Treated the cells as indicated in figures. For the apoptosis cells experiments, collected the supernatant (floating apoptotic cells) and trypsinized the adherent cells from each plate. Wash the collected cells twice with PBS and centrifuge (1000 × rmp, 5 min, RT). For the cell cycle experiments, collected the adherent cells from each plate and fix cells with 70% ethanol for overnight at 4 ℃. The ratio of apoptosis cells and the distribution of cell cycle was performed according to the manufacturer’s protocol FITC Annexin V Apoptosis Detection Kit I (BD, Catalog 556547) and DNA reagent kit (BD, Catalog 340242), respectively. CytExpert software was used for analysis of the results.

### EdU assay

Cells were seed (2 × 10^5^cells) in a 6 well plate 1 day before the experiment, then cells were treated by 100 μM TBHP-treated 1 h and allowed to recover for 24 h. Replaced with medium with EdU labeling solution (final EdU concentration of 10 µM) and incubated cells under appropriate growth conditions for two hours. The EdU positive cells was examined using the BeyoClick^™^ EdU Cell Proliferation Kit with Alexa Fluor 555 (Beyotime, C0075S). The images were acquired using the Imager fluorescence microscope (Nikon, DS-Qi2) utilizing a 20 × objective lens. At least 100 cells for each condition were analyzed and counted for plotting and statistical analyses.

### Xenograft studies

All animal experiments were performed with the approval of Animal Care and Use Ethics Committee of Army Medical Center of PLA. (i) HeLa NC and HeLa shAPE1 cells (ii) HeLa WT cells (5 × 10^6^ in 100 ul medium with Matrigel) were injected subcutaneously in flank of 8 week-old male nude mice. Subcutaneous tumors were allowed to grow for 2 weeks before treatments. When palpable tumors were visible, mice of (i) were divided into four and (ii) were divided into seven groups (n = 7 in each group). Mice were received treatments for 9 days (Ku55933, 10 mg/kg, intraperitonially, daily; E3330, 40 mg/kg intraperitonially, daily; Inhibitor III, 20 mg/kg, intraperitonially, daily; IR, 5 Gy, every 2 days). Specifically, Mice were treated with (i) vehicle, Ku55933, IR, Ku55933 + IR (ii) vehicle, IR, E3330 + IR, Inhibitor III + IR, Ku55933 + IR, E3330 + Ku55933 + IR, and Inhibitor III + Ku55933 + IR. Unblinded tumor measurements were recorded once every 2 days and the volume as calculated by the formula: (width) 2 × length/3. The mice were euthanized in a gas canister with gradual fill carbon dioxide at the end of the treatment cycles and sacrificed. Weighed the tumor with electronic scale.

### Statistical analysis

Each experiment was performed at least three times. Data are expressed as the mean ± SD. The unpaired T test was used to determine differences between means of groups. The difference in overall survival between groups was evaluated with Kaplan- Meier curves and log rank test. A *p *value of < 0.05 / < 0.01/ < 0.001/ < 0.0001denoted by an asterisk */**/***/****.

## Results

### APE1 initiates DSBs generation at the early phase (within 1 h) following IR exposure

**Fig. 1:**
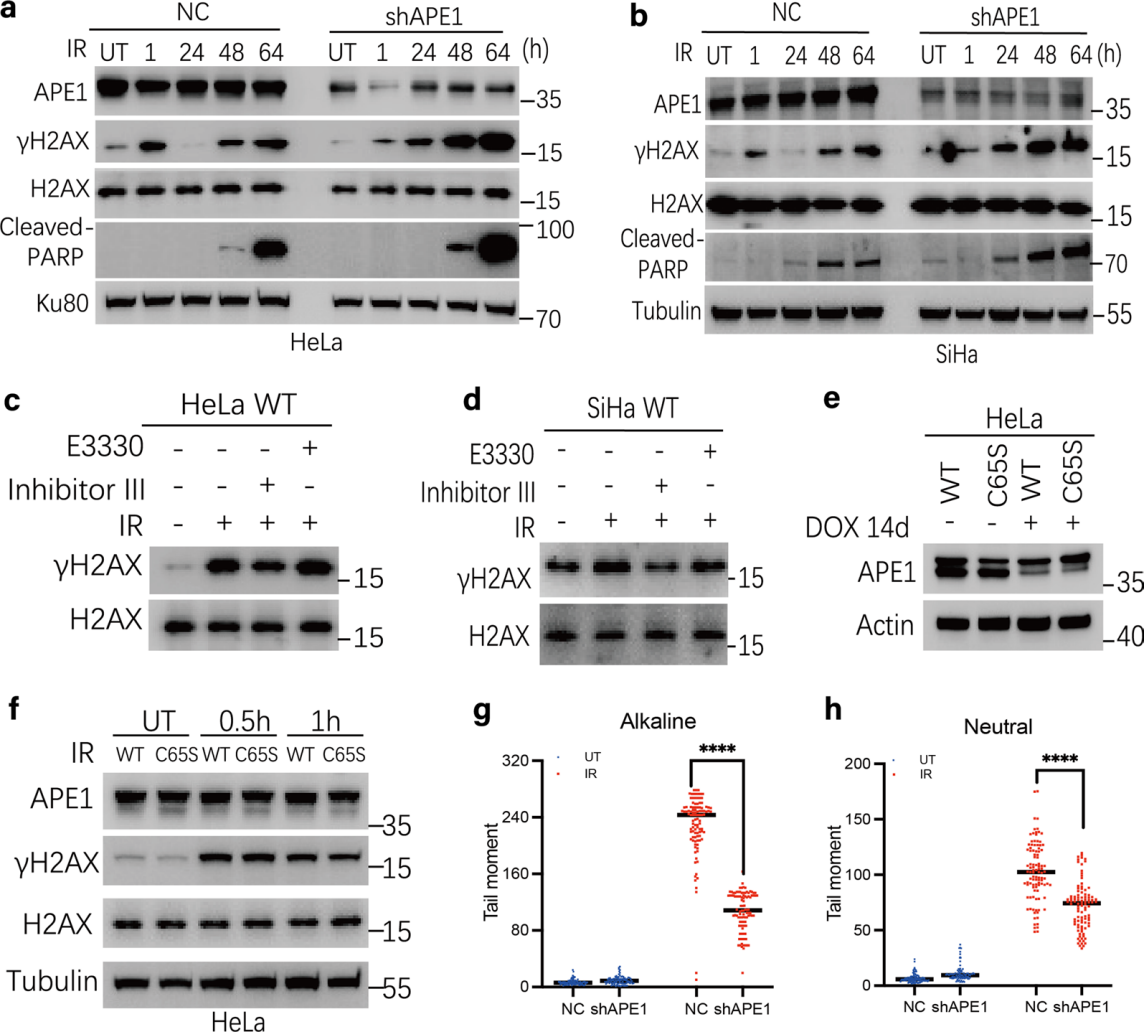
APE1 initiates DSBs generation at an early phase following IR exposure. **a**–**b** APE1 affected temporal formation of DSBs following IR Exposure. NC and shAPE1 cells were treated with 10 Gy IR (treatment for IR was used throughout the study unless stated otherwise) and allowed to recover for various time from 1 to 64 h in HeLa **a** and SiHa **b** cell line. The γ-H2AX and cleaved-PARP level were assayed by immunoblotting. **c**–**d** The endonuclease capacity of APE1 involved in DSBs formation at early phase following IR exposure. HeLa **c** or SiHa **d** WT cells were pre-incubated for 6 h with E3330 (10 μM) or Inhibitor III (2.5 μM) prior to IR treatment and allowed to recover for 1 h. **e** Inducible HeLa APE1^shRNA^ cells were stably transfected with the expression vector encoding wildtype APE1 (WT) or redox activity mutant of APE1 (C65S), respectively. APE1 levels in both cells pre- and post 14-day induction by doxycycline (Dox) were measured by immunoblotting. **f** The γ-H2AX was independent on the redox activity of APE1 at early phase post-damage. HeLa WT-APE1 and HeLa C65S-APE1cells were treated with IR then allowed to recover for 0.5 h or 1 h. **g**–**h** SSB and DSB level of HeLa NC and shAPE1 cell line was evaluated using alkaline and neutral Comet assay, respectively. Cells were treated with IR and allowed to recover for 1 h, followed by comet assays. Tail moment values were calculated for > 100 cells and plotted via a distribution dot plot. The data were presented as the mean ± SEM from three independent experiments. The *p-*values were determined using an unpaired Student’s *t*-test (****p < 0.0001)

We used two different lentivirus-mediated short hairpin RNAs (shRNA) against APE1 to knockdown its expression in the human cervix carcinoma cell line HeLa and SiHa. From here on, APE1 lentiviral negative control and APE1 knockdown cell lines are abbreviated as NC and shAPE1, respectively. In our initial experiments, towards comprehensive investigation of the role of APE1 in DSB formation and repair, we examined DSB levels by measuring γH2AX expression [23] in HeLa and SiHa cell lines with a time-course western blot assay post 10 Gy X-ray irradiation (Fig. 1a, b). In line with previous research, a higher γH2AX signal was observed in shAPE1 cells compared to NC cells at the late time points (24 h/48 h/64 h) post-IR exposure. Conversely, we observed that, at the early time point (1 h), the γH2AX level in shAPE1 cells was markedly lower compared to NC cells. A reduced γH2AX signal was also observed in shAPE1 cells at the early time point (1 h) after treatment with IR in a dose-dependent manner (Additional file 1: Fig S1a, b). Similar DSB results were seen with another shRNA against APE1 (#7958), where even greater KD of the protein was observed (Additional file 1: Fig S3e). These data collectively suggest that APE1 is required for DSB generation post-IR treatment at the early time phase (within 1 h).

As APE1 is a dual function protein, having both redox regulatory and endonuclease activity, we assessed which of these functions was responsible for DSB formation at early phase by pretreating unmodified HeLa and SiHa cells with either a redox inhibitor (E3330, 10 μM) or an endonuclease inhibitor (Inhibitor III, 2.5 μM) for 6 h. As shown in Fig. 1c, d, the γH2AX signal was attenuated only when the endonuclease activity of APE1 was inhibited, suggesting that this capacity of APE1 is responsible for the formation of DSBs. We further excluded the involvement of the redox activity of APE1 in DSB formation by utilizing a redox active site mutant of APE1, C65S-APE1 (Fig. 1e). C65S-APE1 caused similar γH2AX induction post-IR exposure as the wildtype (WT) APE1 expression vector (Fig. 1f). To validate the findings above, we performed alkaline and neutral Comet assays to specifically assess alkaline labile lesions (mostly AP sites and strand breaks) or DSBs, respectively. As shown in Fig. 1g, h and Additional file 1: Fig S1c, the shAPE1 cell line has significantly less SSBs and DSBs compared to NC cells at 1 h post-IR treatment (*p* < 0.001), as measured by comet tail moment, in line with APE1 being responsible for DSBs formation at the early phase post-IR stress.

### APE1 affects temporal DSBs formation following t-butyl hydroperoxide (TBHP) exposure

**Fig. 2:**
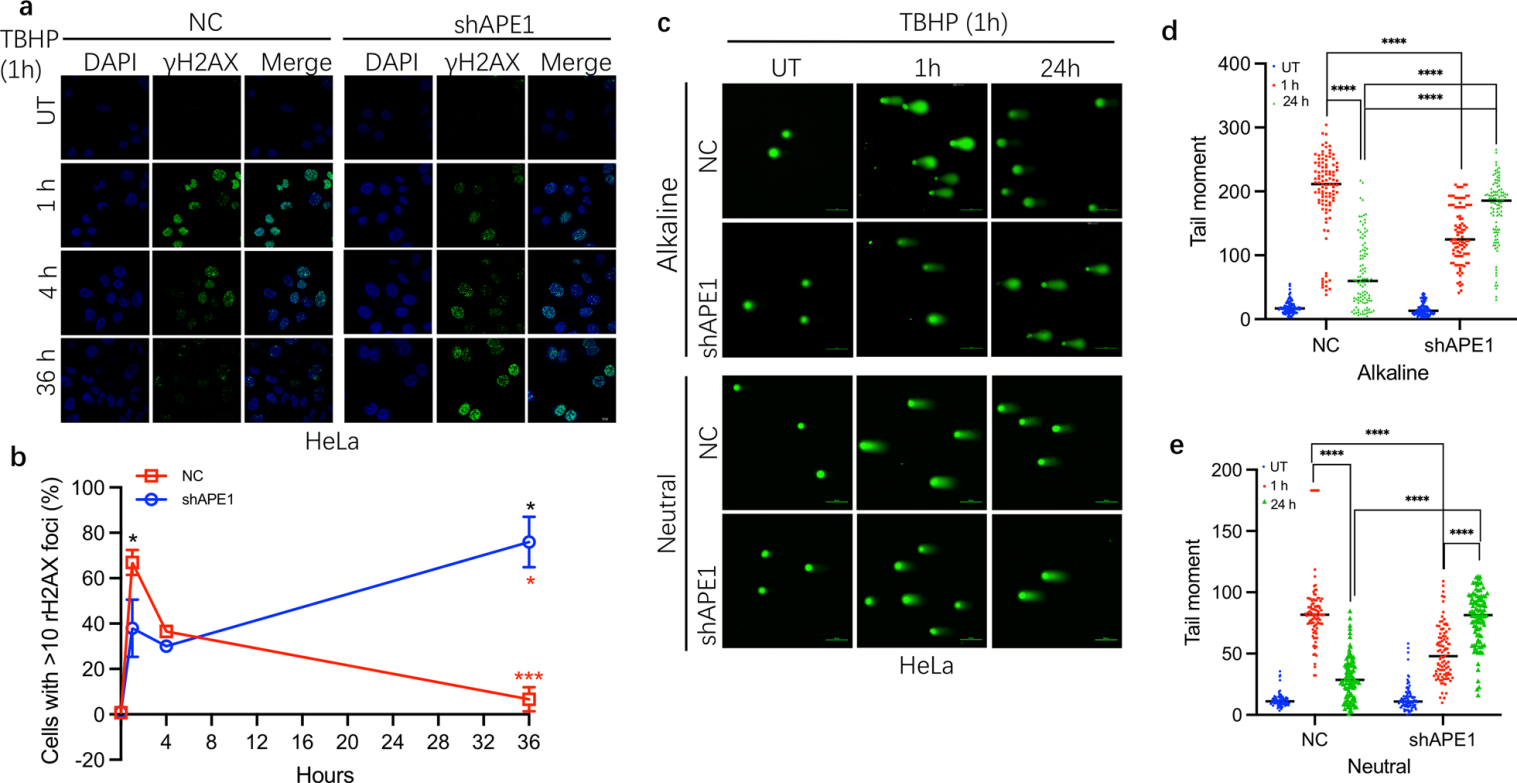
APE1 temporally affects the formation of DSBs following TBHP Exposure. **a** HeLa NC and shAPE1 cell lines were treated with 100 μM TBHP for 1 h (treatment regime for TBHP was used throughout the study unless stated otherwise) and allowed to recover for 1, 4 and 36 h, the distribution of γ-H2AX foci were assessed by IF. **b** the data of **a** were normalized to the proportion of cells with > 10 foci after mock or TBHP treatment. **c**–**e** Residual SSB and DSB levels of HeLa NC and shAPE1 at 1 and 24 h post-TBHP treatment were evaluated using alkaline and neutral comet assays. The representative images are shown in **c**. **d**–**e** Tail moment values were calculated and plotted via a distribution dot plot for > 100 cells. The data were presented as the mean ± SEM from three independent experiments. The *p-*values were determined using an unpaired Student’s *t*-test (****p < 0.0001)

Since the damage induced by IR is mainly derived by reactive oxygen species (ROS) attack following the radiolysis of water. To validate whether the effect of APE1 initial the DSB formation is widespread in oxidative damage, t-butyl hydroperoxide (TBHP), an common oxidizing agent, was utilized to induce oxidative stress and it can also mimic the aspects the oxidative stress of IR [24, 25]. The number of γH2AX foci formed per cell nucleus has been shown to closely correspond to the number of DSBs [26], we examined DSB formation by measuring γH2AX foci in NC and shAPE1 cell line. Consistence with the IR results, γH2AX foci were significantly reduced at 1 h (*p* = 0.021) but increased at 36 h (*p* < 0.001) in APE1-deficient cells compared to NC or scramble cells treated with TBHP (Fig. 2a, b, Additional file 1: Fig S2a). To evaluate whether APE1 deficiency is associated with TBHP-induced DSBs, we performed alkaline and neutral Comet assays. As shown in Fig. 2c–e, TBHP-induced alkaline labile lesions and DSBs were significantly less in the shAPE1 cell line compared to NC cells at the early time point (1 h) but markedly higher at the late time point (24 h), all* p* value is less than 0.001. Combined, our data suggested that APE1 deficiency significantly inhibited DSBs formation at early phase but resulted in numerous DSBs accumulation at late phase in shAPE1 cells following oxidative damage exposure.

### APE1 is required for the activation of DDR and NHEJ repair at the early phase after oxidative stress

**Fig. 3:**
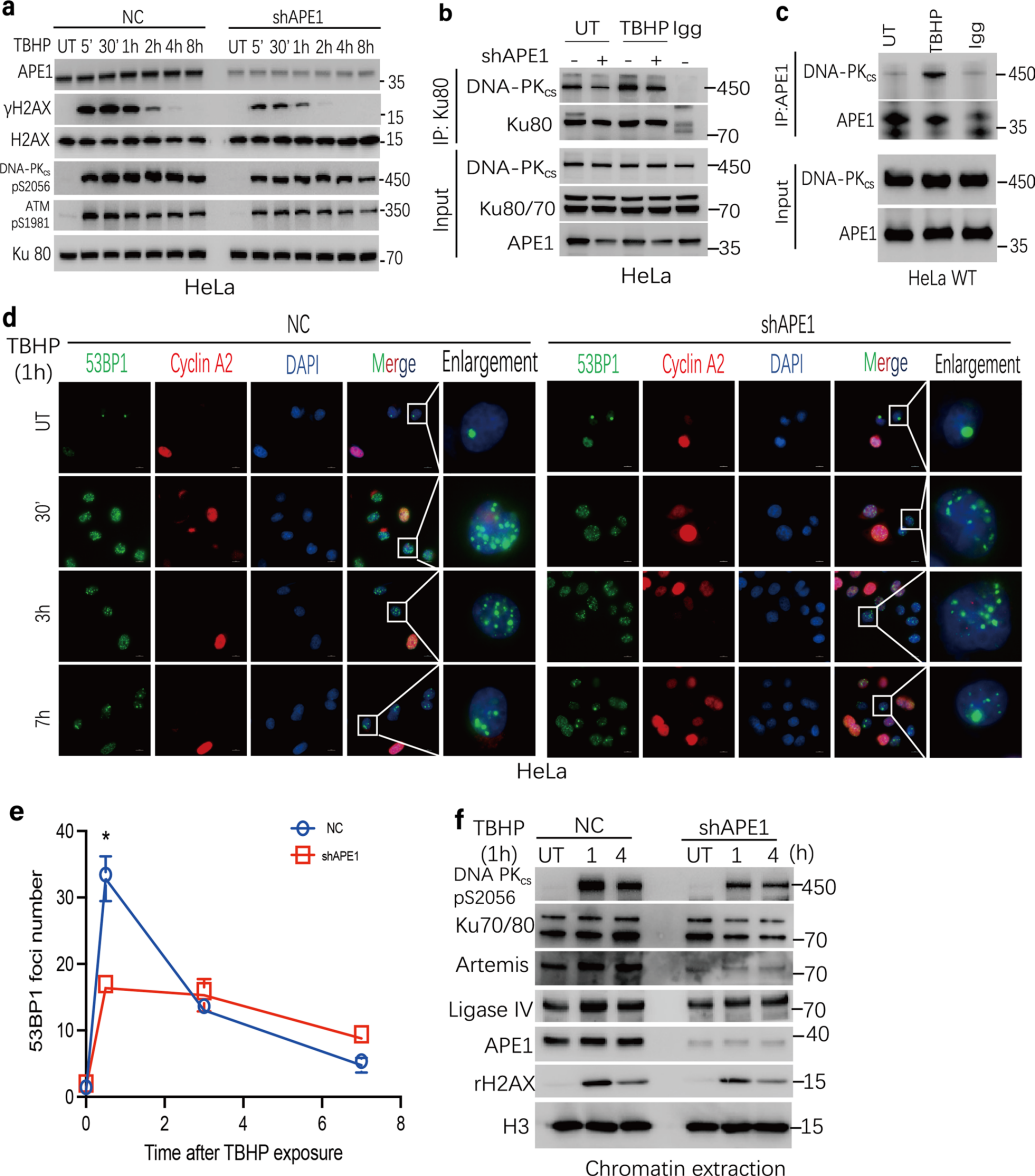
APE1 participates in the DDR and NHEJ repair after oxidative stress. **a** HeLa NC and shAPE1 cells were treated with TBHP for 1 h and allowed to recover for 5 min to 8 h. The γH2AX, DNA-PK_cs_ pS2056 and ATM pS1981 level were assayed by immunoblotting and Ku80 was used as a loading control. **b** The interaction between Ku80 and DNA-PK_cs_ is decreased in APE1 deficiency cells. Ku80 was immunoprecipitated from HeLa NC and shAPE1 cell lines 1 h after TBHP treated 1 h. The immunoprecipitates were analyzed by immunoblotting with anti-DNA-PK_cs_, Ku80 antibodies. **c** The interaction between APE1 and DNA-PK_cs_ in HeLa WT cells, assayed by APE1 immunoprecipitation, was significantly increased post- TBHP treatment 1 h and allowed to recover 1 h. **d**–**e** HeLa NC and shAPE1 cells were treated with TBHP, the resolution of 53BP1 foci were assessed at 0.5, 3 and 7 h post-TBHP treatment. **d** The representative images are shown. **e** The statistical analysis of 53BP1 foci per nucleus are shown. **f** The recruitment of NHEJ factors to the chromatin after TBHP is attenuated in HeLa shAPE1 cells. HeLa NC and shAPE1 cells were treated with TBHP and allowed to recover for 1 and 4 h. Subsequently, the chromatin-enriched fractions were isolated for immunoblotting to assess the recruitment of key NHEJ proteins to chromatin. The *p-*values were determined using an unpaired Student’s *t*-test (*p < 0.05). The data are presented as the mean ± SEM from three independent experiments

Compared to shAPE1 cells, although the DSBs level increased quickly in the early phase in NC cells (within 1 h; see above), it drastically decreased at 24 h post-IR or TBHP exposure, indicative of an efficient DNA repair process. We postulated a novel therapeutic resistant mechanism that DSBs formation initiated by the endonuclease activity of APE1 at the early phase post-oxidative damage is a prerequisite for the subsequent repair process. DDR is complex mechanism to cope with DNA damage, DSBs can trigger cellular DDR signaling to help cells recover from DNA damage [27]. To test this idea, we examined the influence of APE1 on DDR signaling post- TBHP exposure. DDR is generally thought to be driven by three members of the phosphatidylinositol-3-kinase-like kinase (PIKK) family: DNA-dependent protein kinase catalytic subunit (DNA-PK_cs_), ataxia telangiectasia- mutated (ATM), and ataxia telangiectasia-mutated and Rad3-related (ATR) [28, 29]. Since ATR is recognized and recruited to single strand DNA, we first focus on the activation of DNA-PK_cs_ and ATM, as indicated by the autophosphorylation of the former at S2056 or the latter at S1981. We observed that the activation of DNA-PK_cs_, was markedly attenuated in APE1-deficient cells post-TBHP challenge (Fig. 3a, Additional file 1: Fig S3e). Furthermore, flow cytometry results indicated that the proportion of G1 phase cells in the NC group was not statistically different from the shAPE1 group (Additional file 1: Fig S3c–d), implying that activation differences in DNA-PK_cs_ are not caused by an uneven cell cycle distributions following TBHP exposure. Meanwhile, a reduced DNA-PK_cs_ pS2056 signal was also observed in shAPE1 cells after treatment with IR in a dose-dependent manner in HeLa and SiHa cell lines (Additional file 1: Fig S3a, b). Collectively, these data implied that APE1 promoted DSBs repair through the processing of clustered DNA damage and involved in mediating a fully functional DDR in response to DSBs.

Since the DNA-PK_cs_ is not only involved in modulating the DDR, but also a key kinase in the NHEJ repair pathway [30]. We hypothesized that APE1 deficiency may attenuate the NHEJ repair activity following oxidative stress. To test this idea, first, we determined the interaction of the DNA-PK_cs_ and Ku in HeLa NC and shAPE1 cells. Effective DNA-PK_cs_ recruitment and activation by DSBs is required to forming a complex with Ku70/80 heterodimer [31]. Co-immunoprecipitation assays showed that the interaction between DNA-PK_cs_ and Ku80 after TBHP exposure was markedly increased in the NC cell line compared to shAPE1 cell line (Fig. 3b). Meanwhile, co-immunoprecipitation assays also revealed that the interaction between APE1 and DNA-PK_cs_ is significantly increased post-TBHP exposure in HeLa WT cells (Fig. 3c), implicating APE1 participates in the NHEJ pathway directly and facilitates the activation of DNA-PK_cs_. Meanwhile, an increased interaction of DNA-PK_cs_ and APE1 was also observed after treatment with IR in HeLa WT cells (Additional file 1: Fig S3f). Second, we assessed the resolution of TBHP-induced tumor protein p53 binding protein 1 (53BP1) foci, a marker for DSB repair via the NHEJ pathway in G1 phase [32]. As shown in Fig. 3d–e, 53BP1 foci resolution was markedly attenuated and delayed in shAPE1 cells compared to NC cells post-TBHP (*p* = 0.02). Finally, to test whether APE1 is required for loading of the NHEJ machinery at DSB sites, chromatin fractions were isolated from NC and shAPE1 cells either pre- or 1 h and 4 h post-TBHP exposure. After TBHP induction, the NHEJ machinery (DNA-PK_cs_, Ku80, Ligase IV, Artemis) was quickly recruited to DNA following damage in NC cells. However, recruitment of each of these factors to chromatin was significantly attenuated in the shAPE1 cell line, suggesting that APE1 is required for the recruitment and/or loading of the NHEJ machinery to damaged DNA (Fig. 3f). Combined, these data support the hypothesis that APE1 activates NHEJ pathway and thereby results in therapeutic resistance via initiating DSBs generation at the early stage following oxidative agents.

### APE1 deficiency leads to increased artemis protein degradation

**Fig. 4:**
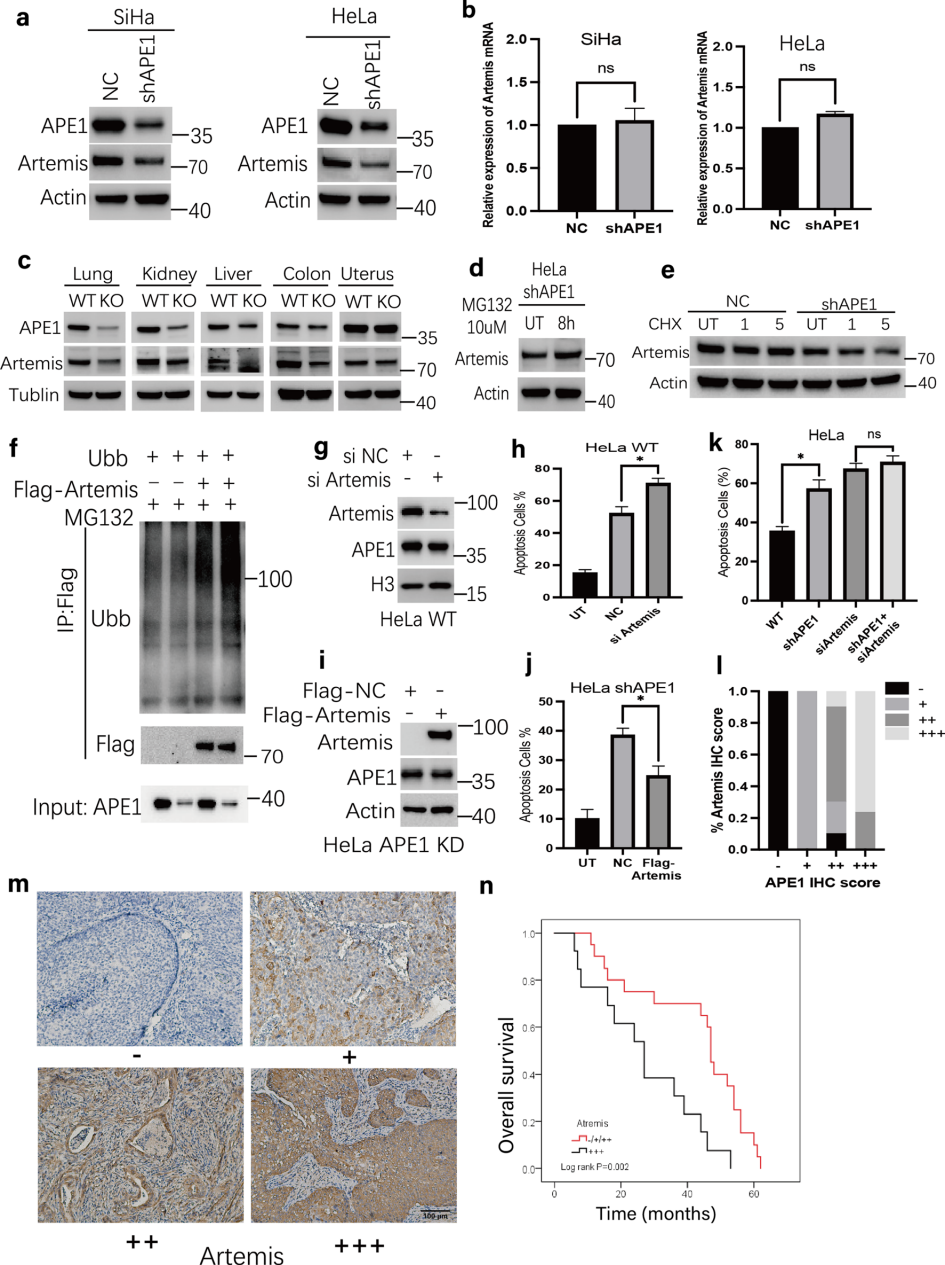
APE1 deficiency leads to increased Artemis protein degradation. **a** Protein expression levels of Artemis in NC and shAPE1 of SiHa and HeLa cells or **c** in the multiple organs in Ape1 WT and KO mice were assessed by immunoblotting. **b** mRNA expression level of Artemis in NC and shAPE1 cells of SiHa and HeLa cells were assessed by qRT-PCR. **d**–**e** HeLa shAPE1 cells were treated with MG132 (10 μM) for 8 h **d**, HeLa NC and shAPE1 cells were treated with cycloheximide (CHX) for 1 and 5 h **e**, then Artemis levels were assayed by immunoblotting. **f** The ubiquitination of Artemis was significantly increased in the HeLa shAPE1 cell line. HeLa ^NC+Ubb^, HeLa ^shAPE1+Ubb^, HeLa ^NC+Ubb+Flag−Artemis^ and HeLa ^shAPE1+Ubb+Flag−Artemis^ cells were treated with MG132. Exogenous expressed Artemis was immunoprecipitated by Flag antibody. And ubiquitination of Artemis was then analyzed by anti-Ubb immunoblotting. **g**, **i** HeLa WT cell line was transfected with si-NC and si-Artemis **g**, HeLa shAPE1 cells were transfected the vector of Flag-NC and Flag-Artemis **i**, the knockdown or overexpression of Artemis was confirmed by immunoblotting. **h**, **j** cells from **g**, **i** were treated with TBHP for 1 h, and apoptosis were analyzed by flow cytometry 48 h later. **k** HeLa WT cell line was transfected with si-NC, si-Artemis, shAPE1 or both, then cells were treated with TBHP for 1 h, and apoptosis were analyzed by flow cytometry 48 h later. **l** The Artemis protein expression was positively correlated with the APE1 protein expression in cervix cancer tissues. **m** The tissue expression of Artemis was evaluated by IHC in 46 cervix cancer patients receiving radical chemo-radiotherapy. Protein levels were scored for four categories: score-, score + , score +  + , and score +  +  + . **n** Kaplan–Meier plot showing different overall survival of cervical cancer patients according to Artemis expression. The *p-*values were determined using an unpaired Student’s *t*-test (*p < 0.05). The data were presented as the mean ± SEM from three independent experiments

The findings mentioned above revealed that APE1 participates in NHEJ repair, we next examined the expression levels of proteins involved in NHEJ. Western blot analyses indicated that the expression of Artemis, an important endonuclease in NHEJ and the repair of radiation-induced DSBs [33, 34], was markedly decreased in APE1-deficient cells (Fig. 4a, Additional file 1: Fig S4b, c). We further examined the Artemis finding in tissue extracts from APE1 WT and knockout (KO) mice, which have been previously reported [35]. Consistent with our result from the in vitro cell experiments, Artemis protein expression is significantly decreased in multiple organs (lung, liver, colon, brain) of the KO mice, in accordance with the APE1 protein levels (Fig. 4c). APE1 deficiency resulted in lower Artemis protein but did not affect Artemis mRNA expression (Fig. 4b), suggesting a specific impact on protein degradation. In addition, the RNA-sequence, between NC and shAPE1 cells, reveals APE1 participates in protein digestion and absorption pathway (Additional file 1: Fig S4a). We therefore investigated whether proteasome inhibition by MG132 could restore the Artemis protein level. In APE1-deficient HeLa cell models, Artemis protein expression was significantly increased by pretreatment for 8 h with MG132 (Fig. 4d). Moreover, exposure of cells to cycloheximide, a general translational inhibitor, resulted in a gradual decreased in Artemis protein levels in shAPE1 cells with similar kinetics to NC cells (Fig. 4e). Further analysis found that the ubiquitination of Artemis significantly increased in shAPE1 cells, implicating a mechanism for targeted protein degradation (Fig. 4f). Meanwhile, a strong positive correlation was observed between APE1 and Artemis protein expression in human cervical cancer samples, with a correlation coefficient of 0.767 (Spearman p < 0.0001) (Fig. 4l). In addition, 46 patients with cervix cancer receiving radical chemo-radiotherapy were used to evaluate whether the expression of Artemis correlate with the clinical outcome of radiotherapy (Fig. 4m, n). The overall survival was longer in the low Artemis group (median OS: 47 m vs 24 m, log-rank* p* = 0.002) than in high group, implying elevated Artemis expression is associated with resistance to radiotherapy.

To determine whether APE1 attenuated the activity of the NHEJ pathway through the downregulation of Artemis protein, we employed siRNA or vector strategies to downregulate or upregulate the expression of Artemis in APE1 WT or deficient cells, respectively (Fig. 4g, h). The results show that siRNA downregulation of Artemis can increase apoptosis of HeLa APE1 WT cells following TBHP exposure (*p* = 0.037), whereas overexpression of Artemis can significantly rescue apoptosis of shAPE1 cells to TBHP, *p* = 0.04 (Fig. 4i, j). Meanwhile, downregulation of both APE1 and Artemis just slightly, but not significantly, induced more apoptotic cell death compared with knockdown of APE1 alone (Fig. 4k; *p* = 0.371). Additionally, an EdU assay, which assesses DNA replication and cell proliferation, revealed that knockdown of APE1 or Artemis individually can inhibit the proliferation of HeLa cells to TBHP exposure, while downregulation of both Artemis and APE1 did not further inhibit cell proliferation (Additional file 1: Fig S4d, e). These data collectively demonstrate that APE1 mediates therapeutic resistance partly via Artemis.

### APE1 is required for cell survival after IR treatment and associated with poor prognosis in cervical cancer patients treated with radiotherapy

**Fig. 5:**
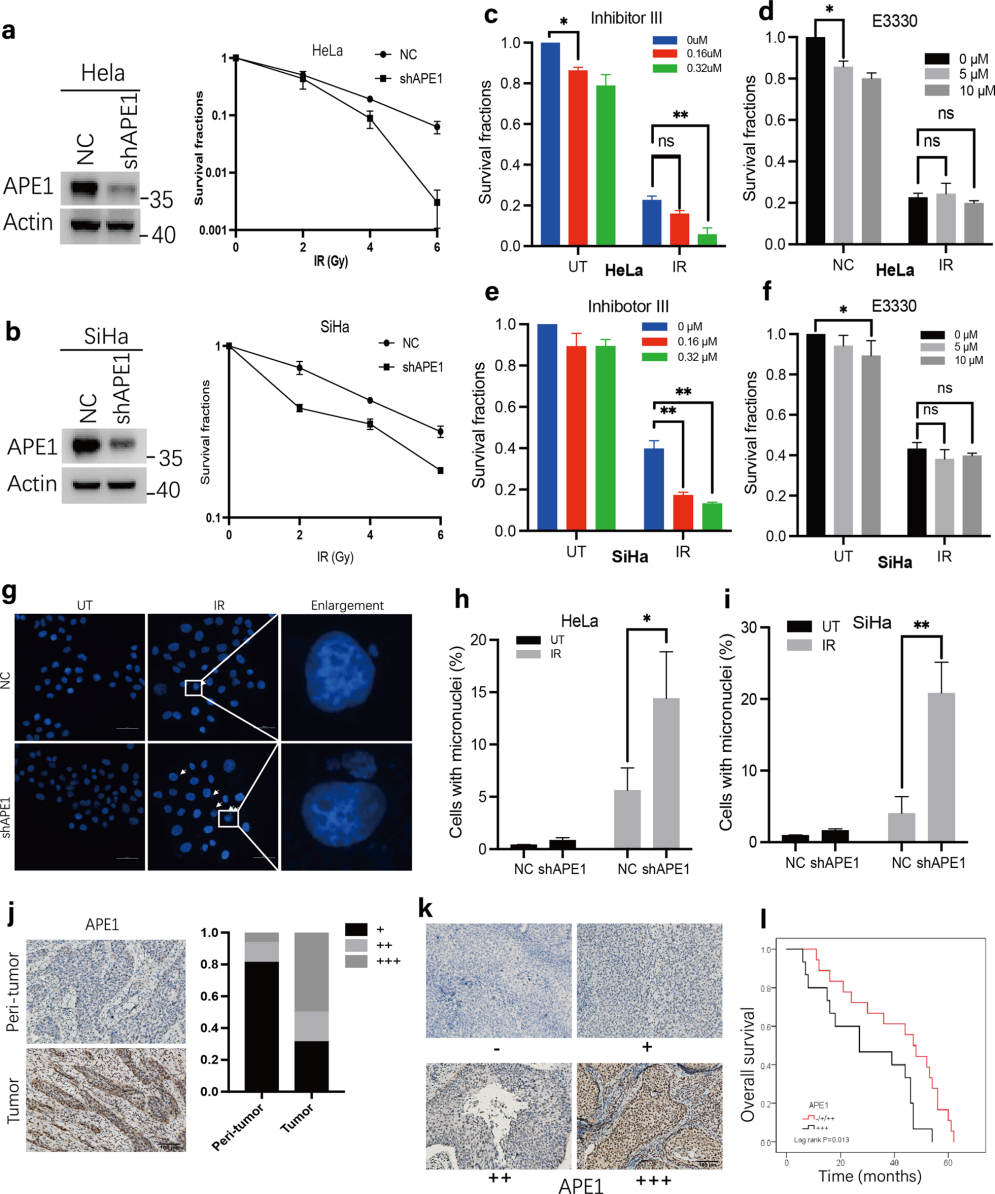
The endonuclease activity of APE1 is required for cell survival after IR treatment. **a**–**b** HeLa and SiHa cell lines were plated and treated with IR at the indicated doses. Colony formation assays were performed to compare sensitivities of the NC and shAPE1 cells. **c**–**f** HeLa and SiHa cells were pre-incubated with Inhibitor III **c**, **e** or E3330 **d**, **f** at the indicated doses for 6 h prior to 3 Gy IR treatment. Colony formation assays were then performed to analysis of survival ability. (**g-i**) NC and shAPE1 cells of HeLa and SiHa cell line were treated with IR, and allowed to recover for 96 h, followed by DAPI staining. The representative graph (HeLa) treated by IR was shown in **g**. **h**–**i** Quantitative analysis of proportion of the number of cells with MNs. **j** The APE1 expression was evaluated by IHC in tumor tissues and paired peri-tumor tissues of 16 cervix cancer samples. The representative images were shown (left) and the bar graph showing the percentage of each score level of APE1 in peri-tumor and tumor tissue, respectively (right). **k**–**l** The tissue expression of APE1 were evaluated by IHC in 46 cervix cancer patients receiving radical chemo-radiotherapy. Protein levels were scored for four categories: score-, score + , score +  + , and score +  +  + . The representative images were shown **k**. Kaplan–Meier plot showing different overall survival of cervical cancer patients according to APE1 expression **l**. The data were presented as the mean ± SEM from three independent experiments. The *p-*values were determined using an unpaired Student’s *t*-test (**p < 0.01, *p < 0.05)

Considering the findings collectively, APE1 promoted NHEJ repair capacity following oxidative damage, then we validate the effect of APE1 expression on cellular response to radiation using a colony formation assay. Consistent with prior literature in human cells [13, 36], our studies indicate that shAPE1 cells (Fig. 5a, b) are more sensitive to IR than the NC cells; the *p* value was 0.024 or 0.004 in HeLa or SiHa cell line treated with 0.3 μM Inhibitor III, respectively. Meanwhile, we also assessed whether the redox or endonuclease capacity of APE1 is involved in the radiosensitivity using colony formation assay. These experiments revealed that the cell survival significantly decreased when the endonuclease activity of APE1 was inhibited but not the redox function (Fig. 5c–f). In addition to apoptosis, mitosis-linked death is another common mode of cell death after irradiation, where cells acquire chromosomal aberrations during division and produce extra-nuclear bodies known as micronuclei. It is generally acknowledged that the major DNA lesion responsible for micronuclei (MN) formation is DSBs [37, 38]. Hence, we measured MN frequency in NC and shAPE1 cell lines in response to IR treatment 96 h post-treatment (Fig. 5g–i). We found that shAPE1 cell markedly increased MN frequency after exposure to IR treatment relative to the NC cells; the *p* value was 0.037 and 0.004 in HeLa or SiHa cell line, respectively. Collectively, the data illustrate that shAPE1 contributes to more MN formation and confers radio-resistance following IR exposure.

We next compared APE1 expression in 16 surgically removed tumor samples and paired peri-tumor tissues of cervix cancer patients by IHC (Fig. 5j). Our data indicate that APE1 is significantly upregulated in tumor samples in comparison to paired peri-tumor tissue (*p* < *0.05*), reinforcing the idea that APE1 is a promising therapeutic target in multiple cancer types. Meanwhile, the result of 46 patients with cervix cancer receiving radical chemo-radiotherapy showed that the overall survival was longer in the low APE1 group (median OS: 45 m vs. 27 m, log-rank *p* = 0.013) group than in high group (Fig. 5k, l). Combined, these data imply elevated APE1 expression is associated with resistance to radiotherapy.

### APE1 deficiency has a synergistic lethal effect with ATM inhibition following oxidative damage exposure

**Fig. 6:**
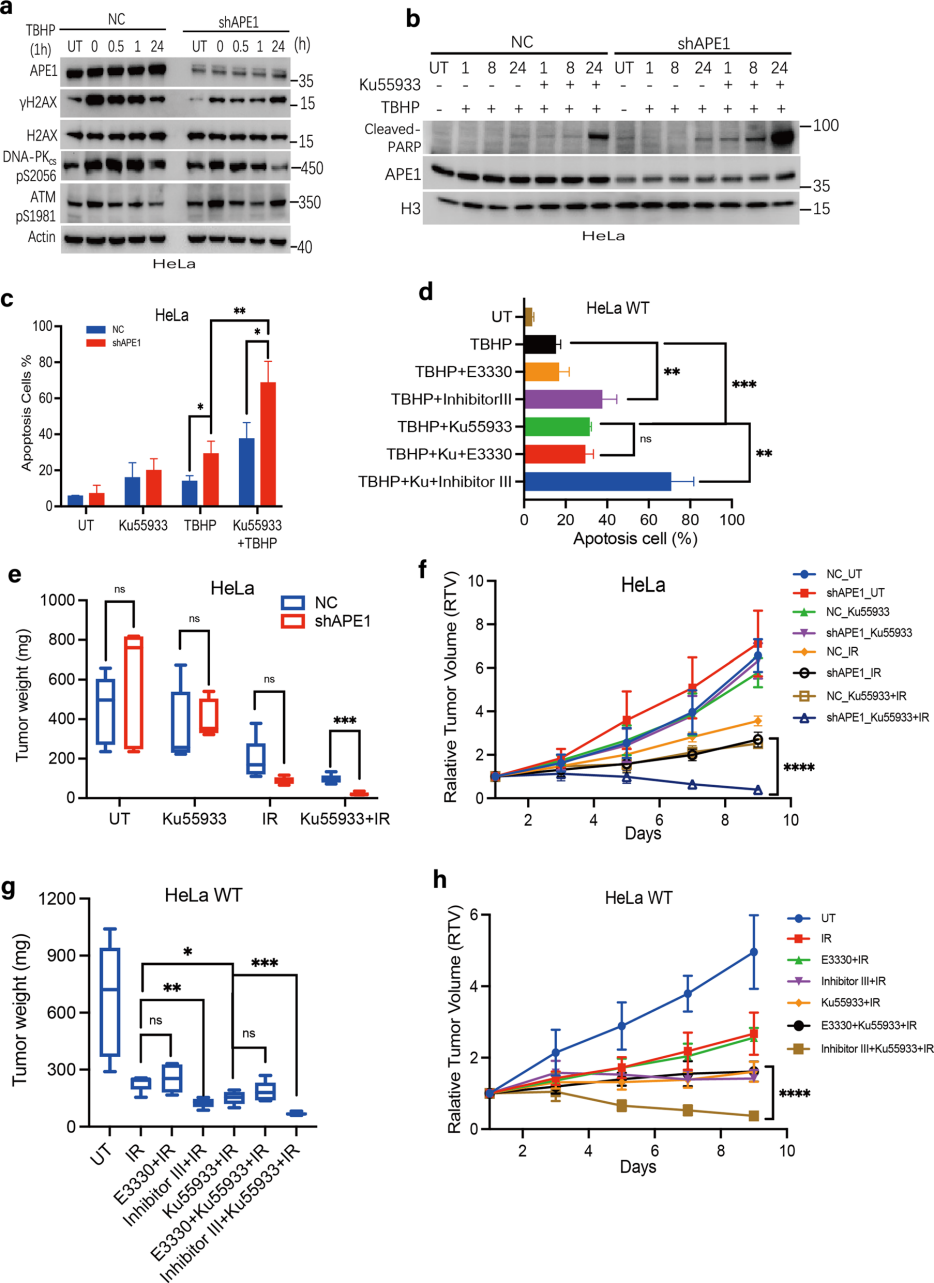
APE1 deficiency sensitizes ATM inhibitor in vitro and suppresses xenograft tumor growth in vivo. **a** HeLa NC and shAPE1 cells were treated with TBHP for 1 h and allowed to recover for 0 h to 24 h. The γH2AX, DNA-PK_cs_ pS2056 and ATM pS1981 level were assayed by immunoblotting and actin was used as a loading control. **b**–**d** APE1 deficiency significantly increased apoptosis when combined with ATM inhibitor (Ku55933). HeLa NC and shAPE1 cells were pre-incubated 6 h with Ku55933 (10 μM) prior to TBHP treatment 1 h **b**–**c**, or HeLa cells were pre-incubated 6 h with E3330, Inhibitor III alone or with Ku55933 prior to TBHP treatment 1 h **d**, allowed to recover for 24 h post TBHP treatment, then the cleaved-PARP was analyzed by immunoblotting **b**; allowed to recover 48 h, apoptosis in each treatment group was analyzed by flow cytometry **c**–**d**. **e**–**f** ATM inhibition sensitized shAPE1 cells to irradiation in vivo. **e** The weights of the resected tumors were quantified. **f** Tumor volume was measured at indicated days and tumor growth curve was plotted. **g**–**h** ATM inhibition sensitized APE1 endonuclease activity inhibitor treated tumor cells to irradiation in vivo. **g** The weights of the resected tumors were quantified. **h** Tumor volume was measured at indicated days and tumor growth curve was plotted. The data are presented as the mean ± SEM from three independent experiments. The *P-*values were determined using an unpaired Student’s *t*-test (****P < 0.0001, ***P < 0.001, **P < 0.01, *P < 0.05)

Considering the findings collectively, APE1 deficiency led to numerous DSBs accumulation at late phase following oxidative damage exposure. DSBs are deleterious DNA lesions that if left them unrepaired. It should be noted, at the late time point (24 h) post-TBHP stress, the γH2AX level in shAPE1 cells was drastically increased compared to NC cells, and the level of ATM pS1981 increased correspondingly (Fig. 6a). This result implied that these late DSBs (at 24 h) still could be partly repair in shAPE1 cells by activate ATM. Hence, to further block the numerous DSBs repair in shAPE1 cells and increasing radio-sensitivity, we examined the synthetic lethal effect of treatment of APE1-deficient cells with an ATM inhibitor (Ku55933) post-TBHP exposure. Flow cytometry assays found that Ku55933 monotherapy has a weak effect on inducing cellular apoptosis in either NC or shAPE1 cells (Fig. 6c). However, when combined with TBHP treatment, Ku55933 significantly enhances cellular apoptosis (Fig. 6b, c), particularly in shAPE1 cells relative to NC cells (*p* = 0.008), suggesting that further impairment of DDR in shAPE1 cells is catastrophic following excessive damage induction. As shown in Fig. 6d, only inhibition of APE1 endonuclease activity exerted a hyper-lethal effect in combination with Ku55933 and oxidative stress (*p* = 0.003). Consistence with the in vitro cell-based results, the addition of Ku55933 significantly inhibited the growth of APE1-deficient xenografts post-irradiation in comparison to control samples (Fig. 6e, f and Additional file 1: Fig S5a), again in an endonuclease-dependent manner (Fig. 6g, h and Additional file 1: Fig S5b); all *p* values were less than 0.01. Collectively, the data indicate that combinatorial inhibition of APE1 and ATM can impart a synergistic lethality in the eradication of cancer cells treated with oxidative stress or IR.

## Discussion

IR induces a range of DNA damage forms. APE1 is generally thought to be involved in radio-resistance by repairing AP sites via the BER pathway. Here, our results demonstrate that APE1 also promotes radio-resistance by initiating the conversion of cluster lesions into DSBs at the early phase (within 1 h) in human tumor cells (Figs. 1, 2). DSBs are highly toxic lesions if left unresolved. However, under the stress of IR or other oxidative genotoxins, the role of DSB formation mediated by APE1 during the early phase on cytotoxicity remains mostly elusive [39]. In *E coli,* the presence of formamidopyrimidine DNA glycosylase (fpg), which contains AP site incision (lyase) activity, is a key determinant in survival following IR exposure [40]. In contrast, in line with previous research, our results indicate that APE1 NC cells are more resistance than shAPE1 cells following IR and TBHP treatment in human cancer cells (Fig. 5). The distinct survival outcome between *E. coli* and human cancer cells under IR stress implies an undefined functional complexity of APE1 in the cellular response.

Compared to shAPE1 cells, although the DSBs level increased quickly in the early phase in NC cells (within 1 h), it drastically decreased at 24 h post-oxidative damage exposure, indicative of an efficient DNA repair process. The ability to repair DSBs plays a critical role in determining the fate of a cell. DSBs are repaired mainly by two mechanisms in organisms: NHEJ and HR [41, 42]. In mammals, both mechanisms are present. However, *E. coli* is incapable of executing classic NHEJ due to the absence of Ligase IV and Ku-like proteins and thus relies on HR to repair DSBs [43, 44]. NHEJ is a primary pathway for the repair of DSBs throughout the cell cycle, and cells or animals containing gene mutations in NHEJ pathway are radio-sensitive. Hence, we postulated that this distinct cytotoxicity between *E. coli* and human tumor cells may in part relate to (i) the different DSB repair capacities and (ii) the role of APE1 in the early phase generation of DSBs and subsequent activation of the DDR and NHEJ repair in human cancer cells post oxidative damage exposure. DNA-PK_cs_ is not only a key kinase in the DDR, but also plays a central role in the NHEJ, a pathway that protects the DNA ends, recruits and activates downstream factors, and promotes efficient end-ligation [45]. In line with our hypothesis, the DDR and NHEJ activity, characterized by the DNA-PK_cs_ pS2056 and the loading of the NHEJ machinery, was significantly increased in the APE1 NC cells compared to the shAPE1 cell line in the early phase post-oxidative damage exposure (Fig. 3).

Our data also indicate that APE1 deficiency impairs DSB repair by attenuating the NHEJ capacity. Evidence includes the observation that APE1 deficient cells and tissue extracts exhibit a markedly decreased expression of Artemis protein (Fig. 4), which is a key nuclease of the NHEJ pathway and functions to repair un-ligatable DSBs [34]. Additionally, the interaction between APE1 and DNA-PK_cs_ significantly increases after oxidative stress, suggesting that APE1 participates in NHEJ repair directly. Combined, besides APE1 playing a role in repair of isolated DNA damage via classic BER, our data indicatd that this protein also modulates therapeutic resistance under oxidative stress by participating in and prompting NHEJ repair. This knowledge is important when considering APE1 as a therapeutic target, particularly when it relates to the combined treatment with a DDR inhibitor. For instance, adding a DNA-PK_cs_ inhibitor before radiotherapy could be a promising alternative for patients with APE1 overexpression by blocking early DSB repair.

Notably, APE1 deficiency leads to DSB accumulation at the late phase following oxidative damage exposure, although during the early phase, the absence of APE1 results in lower levels of DSB formation, likely due to the lack of APE1 processing of induced or BER-generated clustered AP sites. We speculate that the increased levels of DSBs seen at the late phase is likely the result of unrepaired isolated DNA lesions and/or non-DSB clustered lesions acting as roadblocks to replication forks and promoting fork collapse and DSBs formation [46, 47]. Additionally, the impaired NHEJ activity seen in APE1 deficient cells likely leads to further DSB accumulation due to reduced damage resolution. Consistently, Seo Y *et.al* found APE1 deficiency leads to γH2AX accumulation at 48 h after IR treatment [48].

It should be noted that the level of ATM pS1981 also increased correspondingly at the late phase. This result implies that the late DSBs in APE1 deficient cells triggers DDR signaling to help cells recover from oxidative damage. Indeed, the combination of APE1 knockdown and ATM inhibition exacerbated cellular sensitivity to oxidative stress (Fig. 6). Targeting DDR pathways is an attractive strategy for overcoming tumor radio-resistance. Notably, a previous study demonstrated that ATM or DNA-PK_cs_ inhibition induces synergistic lethality in APE1 deficient cells [49], presumably by further down-regulation of DSB repair functionality. Both ATM and DNA-PK_cs_ are indeed key kinases in the DDR and have some overlapping functions [28]. In particular, ATM is activated when it is recruited to DSBs by nijmegen breakage syndrome (NBS1), which functions to promote HR in G2 and S phase [50]. DNA-PK_cs_ is activated when it is recruited to DSBs by Ku, which functions to promotes NHEJ mainly in G1 phase [51]. APE1 deficiency confers radio-sensitization by partially impairing the NHEJ activity (Fig. 3), so there is relatively little room for inducing synergistic lethality in APE1 deficient cells by combining a DNA-PK_cs_ inhibitor. Our data indicate that an ATM inhibitor combination might be a better therapeutic paradigm in APE1 deficient cancer cells under the oxidative damage stress, largely due to the already defective NHEJ activity. Several ongoing clinical trials are investigating the therapeutic effect of ATM inhibitors as part of a palliative treatment combination with radiotherapy [52, 53]. Our data indicate that patients with low expression of APE1 may benefit more from this combinatorial strategy of ATM inhibitor and IR via a synergistic lethal effect. Since DSB formation in the case of APE1 deficiency is relatively late (due to the loss of its endonuclease activity), the timing of administration of the ATM inhibitor should be considered carefully to achieve the most effective synergistic lethality following radiotherapy.

While our study provided new insights into the role of APE1 in the generation and processing of oxidative genotoxic stress-induced DNA damage, there are important limitations to point out. First, APE1 is a multifunctional enzyme, although we observed that only the endonuclease activity of APE1 contributed to the initiation of the DSBs in tumor cells, mouse models designed to inactivate different functions of Ape1 is needed to further verify this finding. Second, compared to the chromatin extraction and 53BP1 recruitment assay in our study, laser micro-irradiation and live cell imaging is a more directly approach to observe the real-time recruitment of NHEJ machinery to DSBs, but we don’t have access to this device currently.

## Conclusion

**Fig. 7:**
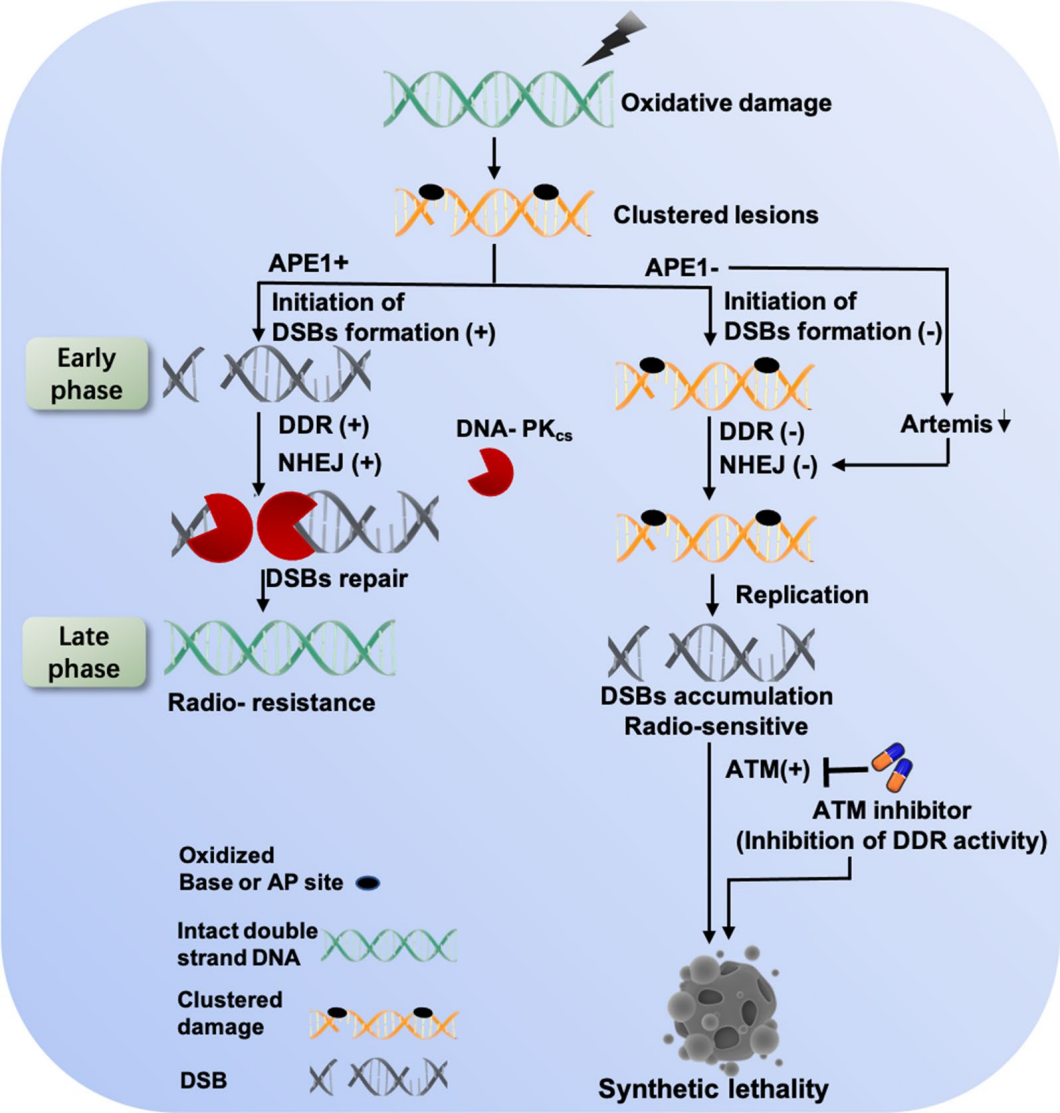
The working model for APE1 contributes to therapeutic resistance following oxidative stress by promoting DDR and NHEJ repair. APE1, via the endonuclease activity, initiates the DSBs formation at the early phase post-oxidative agent exposure, which is a prerequisite for the activation the subsequently DDR and NHEJ repair. In addition, APE1 deficiency attenuates NHEJ capacity by increasing the ubiquitination and degradation of Artemis. Overall, APE1 deficiency results in numerous DSBs accumulation and triggers the activation of ATM at the late phase, inhibition of the ATM significantly promotes synergistic lethality with oxidative damage in APE1-deficient cells

Taken together, our study reveals a novel therapeutic-resistant mechanism involved in APE1 following oxidative genotoxic stress (Fig. 7). We postulate that APE1’s role in radio-resistance by initiating DSBs formation has been missed is due to the generally thought that APE1 simply involves in repairing AP sites to decrease DSBs accumulation by BER pathway. Looking forward, future studies should explore more extensively the effects of timing of inhibitor application with respect to IR administration, perhaps initially using cell models before moving to more complex in vivo tumor models. Additionally, studies of interest would also include whether this finding exists in other treatments inducing oxidative stress, such as chemotherapy and targeted therapy.

## Supplementary Information

**Additional file 1.** **Figure S1**. APE1 involves in DSBs formation at early phase following IR stress. (**a**–**b**) HeLa or SiHa NC and shAPE1 cells were mock-treated or treated with dose-depended IR as indicated in figures and allowed to recover for 1 h. Whole cell lysates were obtained and immunoblotting was performed to assess the γ-H2AX. H2AX and tubulin or actin was used as a loading control. (**c**) Representative images of Figure 1H-I were shown. **Figure S2**. APE1 involves in DSBs formation at early phase following TBHP exposure. HeLa scramble and shAPE1(7958) cell lines were treated with 100 μM TBHP for 1h and allowed to recover for 1, 8, 16 and 36h, the distribution of γ-H2AX foci were assessed by IF. **Figure S3**. APE1 involves in DNA damage response following genotoxic stress. (**a**–**b**) IR induced DDR are attenuated in the APE1 deficient cells. HeLa or SiHa NC and shAPE1 cells were mock-treated or treated with dose-depended IR as indicated in figures and allowed to recover for 1 h. Whole cell lysates were obtained and immunoblotting was performed to assess the DNA-PKcs pS2056, ATM pS1981, KAP1 pS824. Actin was used as a loading control. (**c**) Cell cycle distribution after TBHP exposure. HeLa NC and shAPE1 cells were mock-treated or treated with TBHP for 1 h and allowed to recover for 1 h. Flow cytometry were performed to analysis of the distribution of cell cycle, representative images of figures were shown. (**d**) The data from (**c**) is presented as mean ± SD from three independent experiments. (**e**) HeLa scramble and shAPE1(7958) cells were treated with TBHP and allowed to recover for various time from 0 h to 48 h. The γ-H2AX, DNA-PKcs, and cleaved-PARP level were assayed by immunoblotting. (**f**) The interaction between APE1 and DNA-PKcs in HeLa WT cells, assayed by APE1 immunoprecipitation, was significantly increased post- IR treatment and allowed to recover 1 h. **Figure S4**. APE1 deficiency leads to increased Artemis protein degradation. (**a**) The top 20 significantly enriched KEGG pathways of the differentially expressed genes (DEG) between HeLa NC cells and shAPE1 cells. (**b**) The quantification of Artemis/actin ratio Figure 4A normalized to the value of NC cell line, respectively. (**c**) Protein expression levels of Artemis in scramble and shAPE1(7658) of HeLa and SiHa cells. (**d**) HeLa WT cell line was transfected with NC, shAPE1, siArtemis, or both, then cells were treated with TBHP for 1h, and proliferation were analyzed by EdU assay 24 h later. (**e**) quantitative analysis (**d**) of proportion of the EdU positive cells. **Figure S5**. APE1 deficiency have a synergistic lethal effect with ATM inhibitor in vivo. (a-b) Resected tumors after completion of treatment (Figure 6e and 6g) are shown.

## Data Availability

The authors confirm that the data supporting the findings of this study are available within the article and the Additional file data.

## Abbreviations

53BP1: P53-binding protein
APE1: Apurinic/apyrimidinic endonuclease 1
ATM: Ataxia-telangiectasia mutated
ATR: Ataxia telangiectasia and Rad3-related
BER: Base excision repair
DDR: DNA damage response
DNA-PK_cs_: DNA-dependent protein kinase catalytic subunit
DSB: DNA double-strand breaks
HR: Homologous recombination
IR: Ionizing radiation
KAP1: KRAB the chromatin remodeling factor KRAB domain- associated protein 1
MN: Micronuclei
NHEJ: Non-homologous end-joining
OGG1: 8-Oxoguanine DNA glycosylase
PIKK: Phosphatidylinositol 3-kinase-related kinase
ROS: Reactive oxygen species
SSB: Single strand break
TBHP: Tert-butyl hydroperoxide
