# Supplementary material for: APE1 promotes non-homologous end joining by initiating DNA double-strand break formation and decreasing ubiquitination of artemis following oxidative genotoxic stress
Source: J Transl Med. 2023 Mar 9;21:183. doi: 10.1186/s12967-023-04022-9 (PMC9997026; doi:10.1186/s12967-023-04022-9)
Supplement: Supplementary file 1 — Additional file 1. Figure S1. APE1 involves in DSBs formation at early phase following IR stress. (a–b) HeLa or SiHa NC and shAPE1 cells were mock-treated or treated with dose-depended IR as indicated in figures and allowed to recover for 1 h. Whole cell lysates were obtained and immunoblotting was performed to assess the γ-H2AX. H2AX and tubulin or actin was used as a loading control. (c) Representative images of Figure 1H-I were shown. Figure S2. APE1 involves in DSBs formation at early phase following TBHP exposure. HeLa scramble and shAPE1(7958) cell lines were treated with 100 μM TBHP for 1h and allowed to recover for 1, 8, 16 and 36h, the distribution of γ-H2AX foci were assessed by IF. Figure S3. APE1 involves in DNA damage response following genotoxic stress. (a–b) IR induced DDR are attenuated in the APE1 deficient cells. HeLa or SiHa NC and shAPE1 cells were mock-treated or treated with dose-depended IR as indicated in figures and allowed to recover for 1 h. Whole cell lysates were obtained and immunoblotting was performed to assess the DNA-PKcs pS2056, ATM pS1981, KAP1 pS824. Actin was used as a loading control. (c) Cell cycle distribution after TBHP exposure. HeLa NC and shAPE1 cells were mock-treated or treated with TBHP for 1 h and allowed to recover for 1 h. Flow cytometry were performed to analysis of the distribution of cell cycle, representative images of figures were shown. (d) The data from (c) is presented as mean ± SD from three independent experiments. (e) HeLa scramble and shAPE1(7958) cells were treated with TBHP and allowed to recover for various time from 0 h to 48 h. The γ-H2AX, DNA-PKcs, and cleaved-PARP level were assayed by immunoblotting. (f) The interaction between APE1 and DNA-PKcs in HeLa WT cells, assayed by APE1 immunoprecipitation, was significantly increased post- IR treatment and allowed to recover 1 h. Figure S4. APE1 deficiency leads to increased Artemis protein degradation. (a) The top 20 significantly enriched KEGG pathway [file 12967_2023_4022_MOESM1_ESM.docx]

**Additional file 1**

**
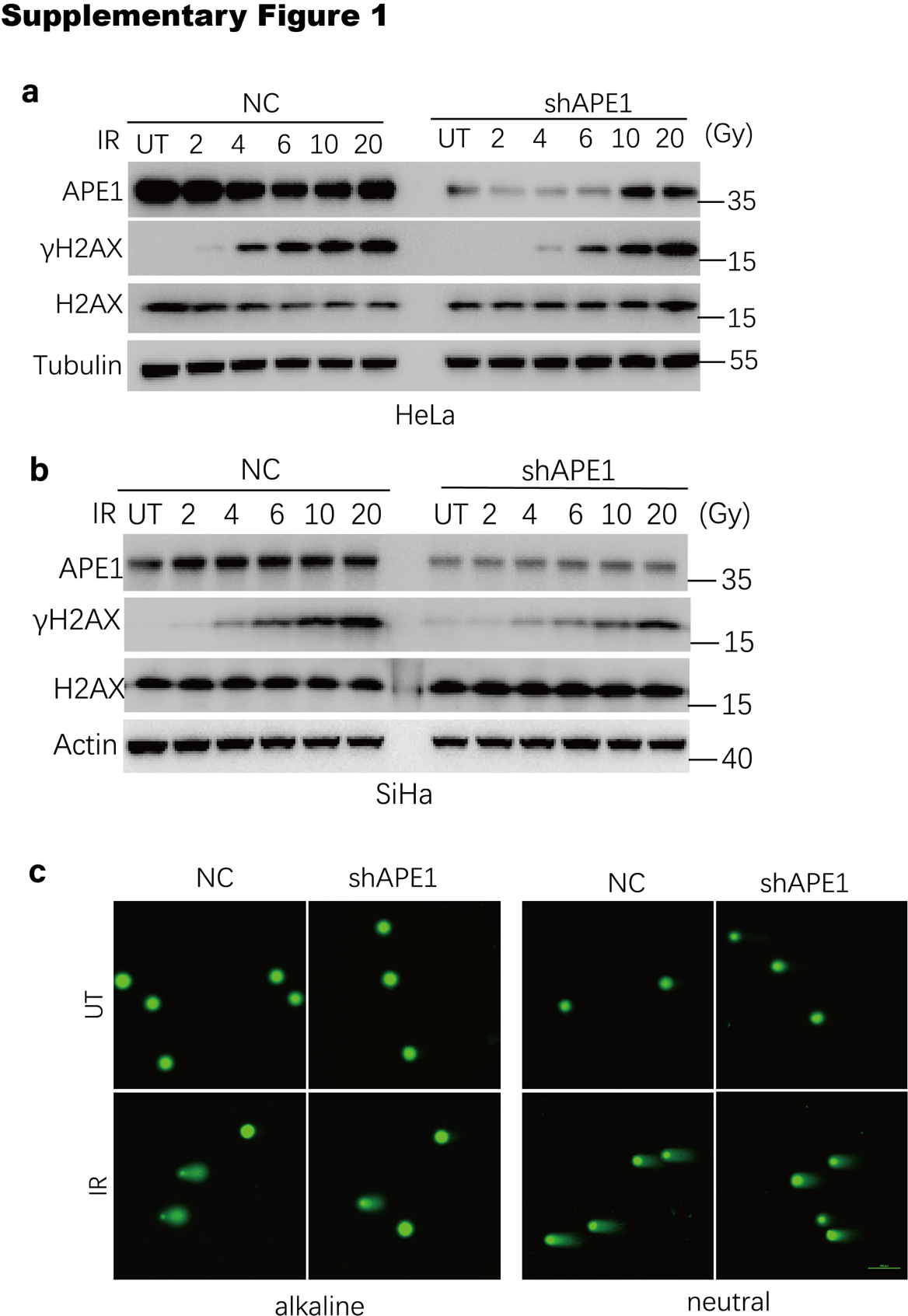
**

**Figure S1.** APE1 involves in DSBs formation at early phase following IR stress.


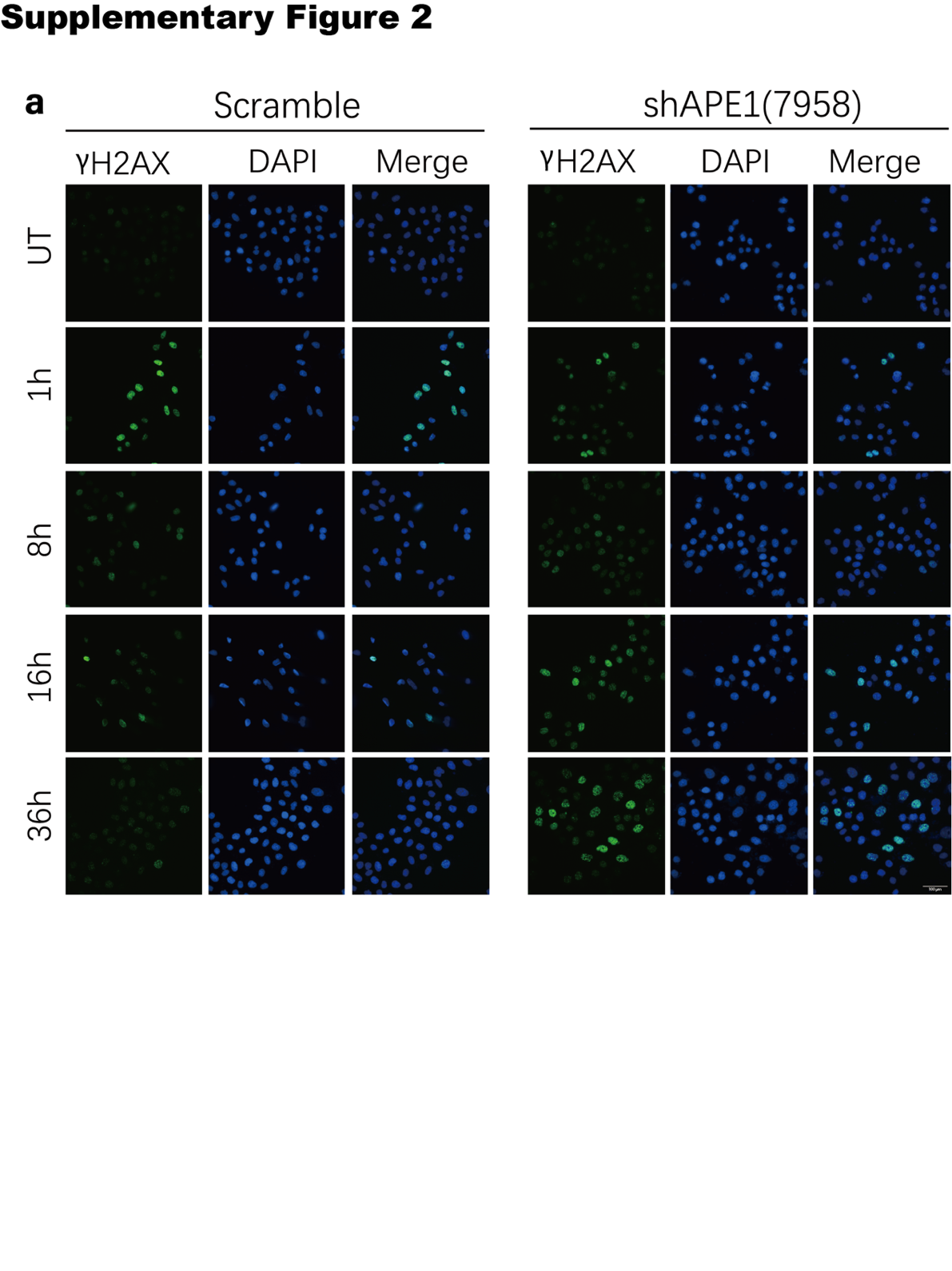


**Figure S2.** APE1 involves in DSBs formation at early phase following TBHP exposure.


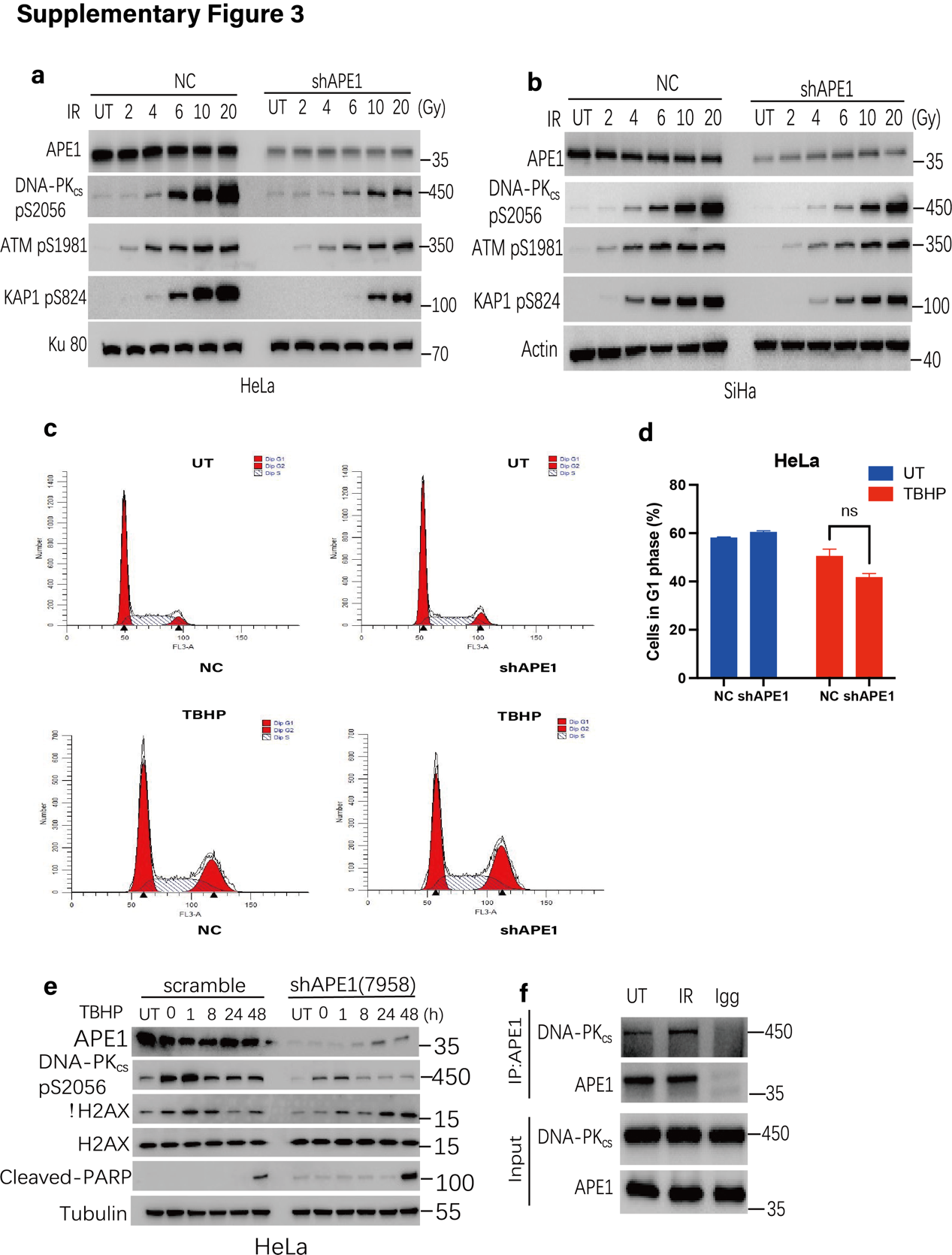


**Figure S3.** APE1 involves in DNA damage response following genotoxic stress.


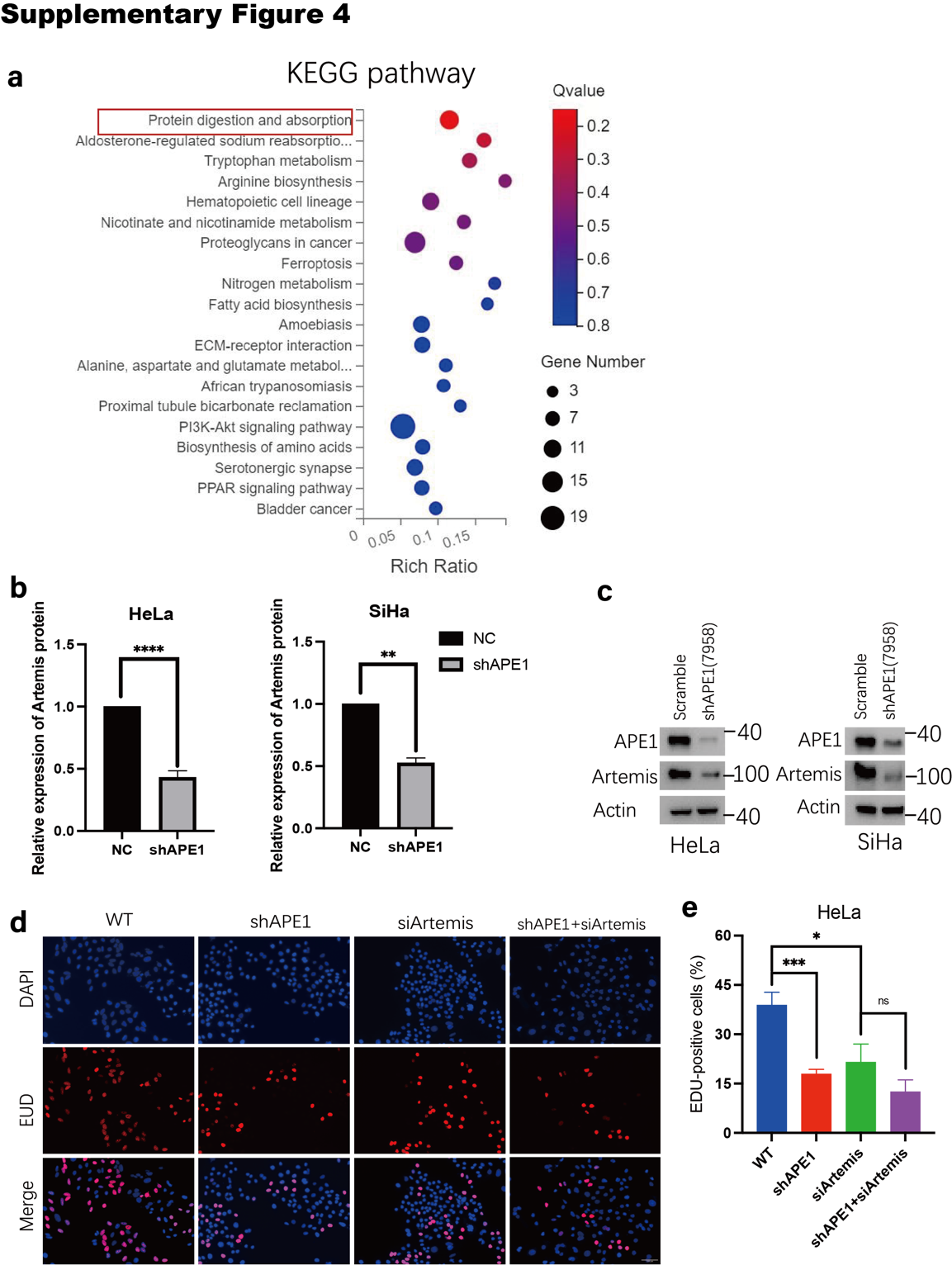


**Figure S4.** APE1 deficiency leads to increased Artemis protein degradation.


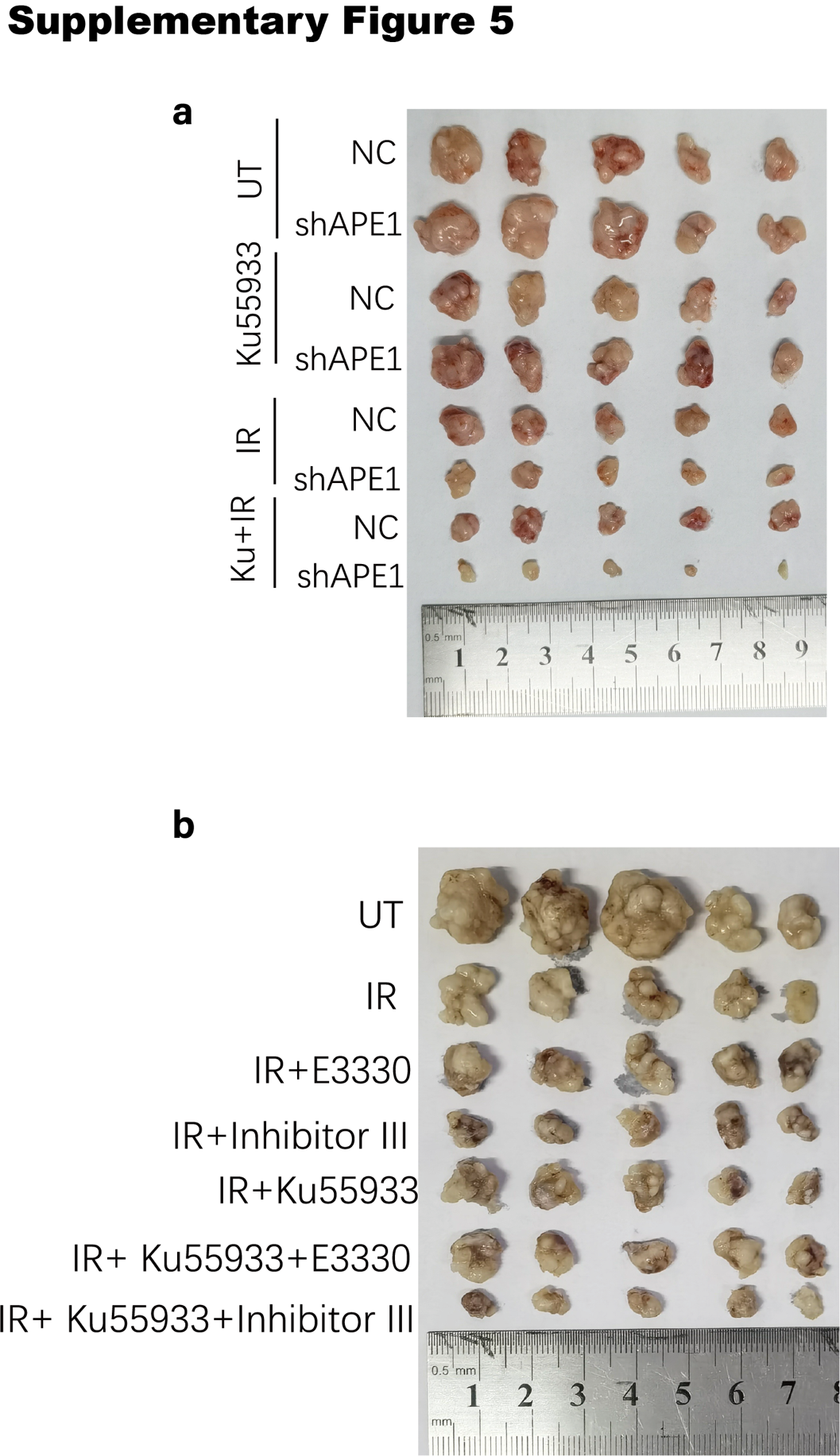


**Figure S5.** APE1 deficiency have a synergistic lethal effect with ATM inhibitor *in vivo*.
